# Whole Genome Sequence Data Provides Novel Insights Into the Genetic Architecture of Meat Quality Traits in Beef

**DOI:** 10.3389/fgene.2020.538640

**Published:** 2020-09-04

**Authors:** Joel D. Leal-Gutiérrez, Fernanda M. Rezende, James M. Reecy, Luke M. Kramer, Francisco Peñagaricano, Raluca G. Mateescu

**Affiliations:** ^1^Department of Animal Sciences, University of Florida, Gainesville, FL, United States; ^2^Faculdade de Medicina Veterinária, Universidade Federal de Uberlândia, Uberlândia, Brazil; ^3^Department of Animal Science, Iowa State University, Ames, IA, United States; ^4^University of Florida Genetics Institute, University of Florida, Gainesville, FL, United States

**Keywords:** cellular structure, connective tissue, energy metabolism, marbling, tenderness and Warner-Bratzler shear force

## Abstract

Tenderness is a major quality attribute for fresh beef steaks in the United States, and meat quality traits in general are suitable candidates for genomic research. The objectives of the present analysis were to (1) perform genome-wide association (GWA) analysis for marbling, Warner-Bratzler shear force (WBSF), tenderness, and connective tissue using whole-genome data in an Angus population, (2) identify enriched pathways in each GWA analysis; (3) construct a protein–protein interaction network using the associated genes and (4) perform a μ-calpain proteolysis assessment for associated structural proteins. An Angus-sired population of 2,285 individuals was assessed. Animals were transported to a commercial packing plant and harvested at an average age of 457 ± 46 days. After 48 h postmortem, marbling was recorded by graders’ visual appraisal. Two 2.54-cm steaks were sampled from each muscle for recording of WBSF, and tenderness, and connective tissue by a sensory panel. The relevance of additive effects on marbling, WBSF, tenderness, and connective tissue was evaluated on a genome-wide scale using a two-step mixed model-based approach in single-trait analysis. A tissue-restricted gene enrichment was performed for each GWA where all polymorphisms with an association *p*-value lower than 1 × 10^–3^ were included. The genes identified as associated were included in a protein–protein interaction network and a candidate structural protein assessment of proteolysis analyses. A total of 1,867, 3,181, 3,926, and 3,678 polymorphisms were significantly associated with marbling, WBSF, tenderness, and connective tissue, respectively. The associate region on BTA29 (36,432,655–44,313,046 bp) harbors 13 highly significant markers for meat quality traits. Enrichment for the GO term GO:0005634 (Nucleus), which includes transcription factors, was evident. The final protein–protein network included 431 interations between 349 genes. The 42 most important genes based on significance that encode structural proteins were included in a proteolysis analysis, and 81% of these proteins were potential μ-Calpain substrates. Overall, this comprehensive study unraveled genetic variants, genes and mechanisms of action responsible for the variation in meat quality traits. Our findings can provide opportunities for improving meat quality in beef cattle via marker-assisted selection.

## Introduction

Consumption studies suggest that quality is more important compared to other factors such as product price in the consumer’ purchasing decision (Henchion et al., 2014). However, despite continuous efforts to ensure high quality, the beef industry experiences a wide range of variability in meat quality, which in turn contributes to consumer dissatisfaction. There are two components to the meat quality perception, an expected and an experienced quality component, and a mismatch between them could be a source of consumer dissatisfaction affecting future willingness to purchase beef (Hocquette et al., 2014).

Tenderness and flavor are two meat quality-related phenotypes identified as the top purchasing motivators in the United States for fresh beef steaks (Reicks et al., 2011). Like all other meat quality attributes, these are complex traits controlled by genetics and influenced to a large degree by environmental factors (Leal-Gutiérrez and Mateescu, 2019). Moreover, they are difficult and expensive to measure on a large number of animals and might require progeny testing which makes them perfect candidates for genomic research aimed at identifying genetic markers accounting for variability in meat quality and ultimately the causative mutations responsible for this variation. These findings would be highly beneficial for the beef industry since consumers are willing to pay a higher price if higher meat quality can be guaranteed (Lyford et al., 2010; Polkinghorne and Thompson, 2010).

Genome-wide association studies are frequently used to identify genomic regions able to explain variation in complex phenotypes. However, the high-density single nucleotide polymorphism (SNP) arrays commonly used in these analyses do not allow the discovery of causative mutations associated with complex phenotypes. In general, genome-wide association (GWA) studies report associated genomic regions which contain several possible candidate genes given the high level of linkage disequilibrium in cattle. Causative variants are more likely to be identified when using whole-genome level sequence for association analyses which allows pinpointing the actual causative polymorphism (Meuwissen et al., 2013). Identification and characterization of causal genetic variants could increase the accuracy and robustness of the genomic selection, ultimately having a greater impact on the genetic improvement of meat quality-related traits (Daetwyler et al., 2014).

The objectives of the present analysis were to (1) perform a GWA analysis for marbling, WBSF, tenderness, and connective tissue using whole-genome sequence data in an Angus population, (2) identify enriched pathways from each GWA analysis; (3) construct a protein–protein interaction network using all the associated genes and (4) perform a μ-Calpain proteolysis assessment for associated structural proteins.

## Materials and Methods

The research protocols were approved by the Iowa State University and Oklahoma State University Institutional Review Boards.

### Population Description

An Angus-sired population of 2,285 individuals was available for this study. This population contained 1,311 steers, 540 bulls and 434 cows progeny from 173 Angus sires. Cattle from Iowa State were fed in a feedlot in Iowa (*n* = 1,085), cattle from Jack Cowley Commercial Angus Ranch in Montague, CA, United States, were all fed in California (*n* = 360), and cattle from Duck Smith Farms in Meridian, OK, were fed either in Colorado (*n* = 388) or Texas (*n* = 452) using a concentrate based diet. Animals were transported to a commercial packing plant and harvested at an average age of 457 ± 46 days and live weight of 532 ± 62 kg. Fabrication of the carcasses was performed according to the Institutional Meat Purchasing Specifications (IMPS; USDA, 1996). From each carcass, rib sections were collected in Iowa, California, and Colorado, and in Texas, strip loins (IMPS #180) were collected. Muscles were labeled, vacuum-packaged, boxed, and transported to the Iowa State University Meat Laboratory, Ames, or the Oklahoma State University (OSU) Food and Agricultural Products Center, Stillwater, for fabrication. Two 2.54-cm steaks were sampled from each muscle for recording of WBSF and sensory analysis. Steaks were vacuum packaged and aged till the 14th-day postmortem at 2°C. After completing aging, all steaks were then frozen at −20°C for subsequent analysis. Steaks fabricated in Iowa were transported to the OSU Food and Agricultural Products Center (Garmyn et al., 2011; Mateescu et al., 2016, 2017).

### Recorded Phenotypes

The phenotypic evaluation performed in this population was described in detail by Garmyn et al. (2011) and Mateescu et al. (2016). A brief description of the phenotypes used in this study are presented.

#### Marbling

Marbling was recorded 48 h postmortem by graders’ visual appraisal of the ribeye muscle at the cut surface after the carcass had been ribbed at the 12th/13th rib interface. The marbling grade was as follows: 3.0 = traces, 4.0 = slight, 5.0 = small, 6.0 = modest, 7.0 = moderate, 8.0 = slightly abundant, 9.0 = moderately abundant, and 10 = abundant.

#### Warner-Bratzler Shear Force

Before processing, all frozen steaks were allowed to thaw at 4°C for 24 h. Steaks were broiled in an impingement oven (XLT Impinger, model 3240-TS, BOFI Inc., Wichita, KS, United States or Lincoln Impinger, model 1132000-A, Lincoln Foodservice Products, Fort Wayne, IN, United States) at 200°C to an internal temperature of 68°C. An Atkins AccuTuff 340 thermometer (Atkins Temptec, Gainesville, FL, United States) was used to monitor the temperature of steaks as they exited the oven when the internal temperature reached 68°C. After cooking, steaks were cooled at 4°C for 18–24 h as recommended by the American Meat Science Association (AMSA, 1995). Six cores with a 1.27-cm diameter and parallel to the muscle fiber were sheared with a Warner-Bratzler head attached to an Instron Universal Testing Machine (model 4502, Instron Corporation, Canton, MS, United States). The Warner-Bratzler head moved at a crosshead speed of 200 mm/min. The average peak load (kg) of the six cores from the same steak was calculated for each steak.

#### Sensory Analysis

Frozen steaks were randomly assigned to each sensory session, and thawed and cooked as described above for WBSF. Cooked steaks were sliced into approximately 2.54 cm × 1.27 cm × 1.27 cm samples, and provided to each panelist. The sensory panel consisted of eight members trained for tenderness and connective tissue attributes (Cross et al., 1978). Sensory sessions were conducted once or twice per day, and 12 samples were evaluated during each session. Samples were evaluated using a standard ballot (AMSA, 1995). Duplicated samples were provided to each panelist to measure tenderness and connective tissue using an 8-point scale in the following way: tenderness (8 = extremely tender, 7 = very tender, 6 = moderately tender, 5 = slightly tender, 4 = slightly tough, 3 = moderately tough, 2 = very tough, 1 = extremely tough) and connective tissue (8 = none detected, 7 = practically none, 6 = traces amount, 5 = slight amount, 4 = moderate amount, 3 = slightly abundant, 2 = moderately abundant, 1 = abundant amount). Panelists were randomly seated in individual booths in a temperature- and light-controlled room. The 12 samples were served in a randomized order by panelists. The panelists were provided distilled, deionized water and unsalted crackers to cleanse their palate. For each steak, the average score from all the panelists was analyzed.

### Genotyping and Whole Genome Sequence Imputation

Genomic DNA was extracted from muscle and genotyped with the Bovine SNP50 Infinium II BeadChip (Illumina, San Diego, CA, United States). SNPs with minor allele frequency (MAF) lower than 5%, and calling rate for sample and SNP lower than 95% were removed. A total of 40,875 SNPs were retained and used for the imputation analysis (Mateescu et al., 2017).

First, all samples at 40,875 SNPs were imputed to 770k genotypes using annotation for the *Bos taurus* UMD 3.1.1 assembly. Following, these genotypes were further imputed to whole genome sequence using 255 Angus bulls from the ISU herd (52 individuals) and the 1,000 bulls genome project (203 individuals; run 4) (Daetwyler et al., 2014). Imputation was done using FImpute (Sargolzaei et al., 2014) and the SNPipeline suite^1^ on a chromosome-by-chromosome basis. The imputation accuracy was approximately 95%.

Once individuals were imputed to the whole genome sequence, there were approximately 44.3 million SNPs that could be used, as opposed to the prior 40,875 SNPs pre-imputation.

### Association Analysis and Assignment of Associated Markers

Genotype data for 44,294,424 SNP markers were available for 2,268 Angus animals with recorded meat quality phenotypes. The SNP markers that mapped to the sex chromosomes or had MAF less than 0.1% were removed from the dataset. Marker allelic frequency was calculated with Plink 1.90b3.39 for each trait, taking into account only animals with records for the trait under evaluation. After quality control, approximately 14.6 million SNP markers were retained for subsequent genomic analysis.

Given the categorical nature of marbling, tenderness and connective tissue, the assumption of normality was previously verified in this Angus dataset by Mateescu et al.(2015). The relevance of additive effects on marbling, WBSF, tenderness, and connective tissue was evaluated on a genome-wide scale using a two-step mixed model-based approach (Aulchenko et al.,2007a,b) in single-trait analyses. In the first step, the following mixed model was fitted

y=X⁢β+Zu+e,

where *y* is the vector of phenotypic records for the trait under evaluation, *X* is the incidence matrix linking fixed effects to phenotypic records, β is the vector of fixed effects including a general intercept, the contemporary group, defined as one character for source (I = Iowa State, C = Cowley, D = Duck Smith), 8 digits for harvest date (YYYYMMDD), and 2 digits for harvest sex (01 = bull, 02 = steer, 03 = cow), as class effect with 33 levels for marbling and WBSF and 20 levels for tenderness and connective tissue with at least 11 up to 213 animals per level, and marbling as covariate for WBSF, tenderness and connective tissue, *Z* is the incidence matrix relating phenotypic records to animal effects, *u* is the vector of random animal effects, and *e* is the vector of random residual effects. The random effects were assumed multivariate normal with $u∼N\,\left(0,G\,\sigma_{\text{u}}^{2}\right)$ and $e∼N\,\left(0,I\,\sigma_{\text{e}}^{2}\right)$, where $\sigma_{\text{u}}^{2}$ and $\sigma_{\text{e}}^{2}$ are the animal additive genetic and residual variances respectively; *G* is the genomic relationship matrix and *I* is an identity matrix. In our case, *G* was created using 50,196 randomly selected SNPs with SNP number per chromosome proportional to chromosome length. The variance-covariance matrix of this model was estimated as $V_{0}=Z\,G\,Z^{\prime }\,\sigma_{\text{u}}^{2}+I\,\sigma_{\text{e}}^{2}$.

In the second step, for the whole-genome scan, the following model was fitted for every SNP, *y* = *X*β + *X*_SNP_β_SNP_ + *e*, assuming $e∼N\,\left(0,V_{0}\,\sigma_{\text{e}}^{2}\right)$, where *X*_*SNP*_ is the design matrix for the marker under study and β_SNP_ is the regression coefficient or SNP effect. The significance of each additive effect was tested using the following test statistic,

$$\text{z}=\frac{X_{\text{SNP}}^{\prime }\,V_{0}^{-1}\,\left(y-X\,\hat{\beta }\right)}{\sqrt{X_{\text{SNP}}^{{}^{\prime }}\,V_{0}^{-1}\,X_{\text{SNP}}}}$$

which approximates the Wald test, and is asymptotically standard normal. These analyses were performed using the R package MixABEL (Aulchenko et al., 2010). Polymorphisms with *p*-value < 1 × 10^–4^ were considered significant and <1 × 10^–7^ as highly significant markers.

Gene annotation for the *B. taurus* UMD 3.1.1 assembly was downloaded from Ensembl (Zerbino et al., 2018) and used to assign significant SNPs to genes using the following criterion: the marker was determined as genic if it was located inside a gene; if the marker was not inside a gene, it was assigned to a gene using the following four marker-gene bin distances: 5, 10, 50, and 100 kb. Associated markers located farther away from a gene were determined as intergenic. Regions harboring intergenic markers were further assessed to determine if they could lay on a gene desert. A gene desert was assumed as a >500 kb nucleotide genomic region where no protein coding gene is described (Schierding et al., 2014). The distance between the associated markers and both adjacent genes had to be higher than 200 kb.

### LD Analysis

Five highly significant regions (*p*-value < 1 × 10^–7^) located on chromosome 29 (BTA29: 36,432,655–44,313,046 bp) were selected to perform an LD-block analysis. The five selected regions were (1) between the base-pair positions 37,062,087 and 37,077,105 (62 SNPs); (2) *PAG18* gene (77 SNPs); (3) *FADS1* and *FEN1* genes (101 SNPs); (4) *GNG3*, *BSCL2*, and *ENSBTAG00000047110* genes (132 SNPs) and (5) *SYVN1* and μ*-calpain* genes (92 SNPs). LD-blocks were constructed using the Haploview software (Barrett et al., 2005) with a confidence interval of minimum of 98% for strong LD (Gabriel et al., 2002), and Hardy–Weinberg equilibrium p-value cutoff and MAF higher than 0.001. All individuals were included as singletons.

### Gene Enrichment Analysis

A tissue-restricted gene enrichment was performed for each GWA. The methodology described by Baranzini et al. (2009) was used and carried out using an in-house JAVA script. From each GWA, all polymorphisms with an association p-value lower than 1 × 10^–3^ were included in the analysis. Each significant marker was assigned to a gene if the polymorphism was located within 3 kb upstream or downstream from a gene or inside a gene. Gene annotation for the *B. taurus* UMD 3.1.1 assembly was downloaded from Ensembl (Zerbino et al., 2018). The tissue restriction was carried out by keeping only genes expressed in bovine skeletal muscle. A total of 10,919 genes reported by the EMBL-EBI Expression Atlas and 8,090 other genes identified as expressed in skeletal muscle in a multibreed Brahman-Angus population (Leal-Gutiérrez et al., 2019) were included. The total number of genes used for this filtering was 13,155, and this gene list was used as background.

The available GO terms for the background list of genes expressed in bovine skeletal muscle were obtained using Biomart from Ensembl (Zerbino et al., 2018). A total number of 96, 106, 95, and 101 GO terms for marbling, WBSF, tenderness and connective tissue, respectively, were included. Gene lists with 1,391, 1,394, 1,385, and 1,443 significant genes for marbling, WBSF, tenderness and connective tissue, respectively, were used. Gene enrichment was conducted using the hypergeometric test available in the Apache Commons Mathematics Library for JAVA.

### Protein–Protein Interaction Network

The genes identified as associated using the highly significant threshold (<1 × 10^–7^) with marbling, WBSF, tenderness, or connective tissue, were selected, and a protein–protein interaction network was constructed using the IntAct Molecular Interaction Database of Ensembl (Orchard et al., 2014). SNPs tagging more than one gene by trait were excluded. The protein–protein interaction network was visualized using Cytoscape 3.7.1 (Shannon et al., 2003). No self-loop was allowed.

### Candidate Structural Protein Assessment of Proteolysis

The genes identified as associated using the highly significant threshold (<1 × 10^–7^) and also encode structural proteins were analyzed for a protease substrate analysis. This analysis was done using the PROSPER server (Song et al., 2012), which reports proteins that are potential substrates of μ-Calpain. Of a total of 83 genes with association *p*-value < 1 × 10^–6^, 42 were structural proteins (integral components of plasma or organelle membranes, cytoskeletal proteins, and organelle matrix-associated proteins) and were included in the assay for proteolysis.

## Results

### Recorded Phenotypes

Table 1 shows descriptive statistics for the meat quality related phenotypes used in the present analysis.

**TABLE 1:** Basic statistics for the meat quality phenotypes.

	*N*	Mean	*SD*	Minimum	Maximum
Marbling	2268	5.97	1.04	3.00	9.80
WBSF (kgs)	2235	3.53	0.77	1.49	8.47
Tenderness	1718	5.80	0.59	3.00	7.38
Connective tissue	1718	5.88	0.60	3.13	7.25

### Association Analysis for Meat Quality Related Phenotypes

**FIGURE 1:**
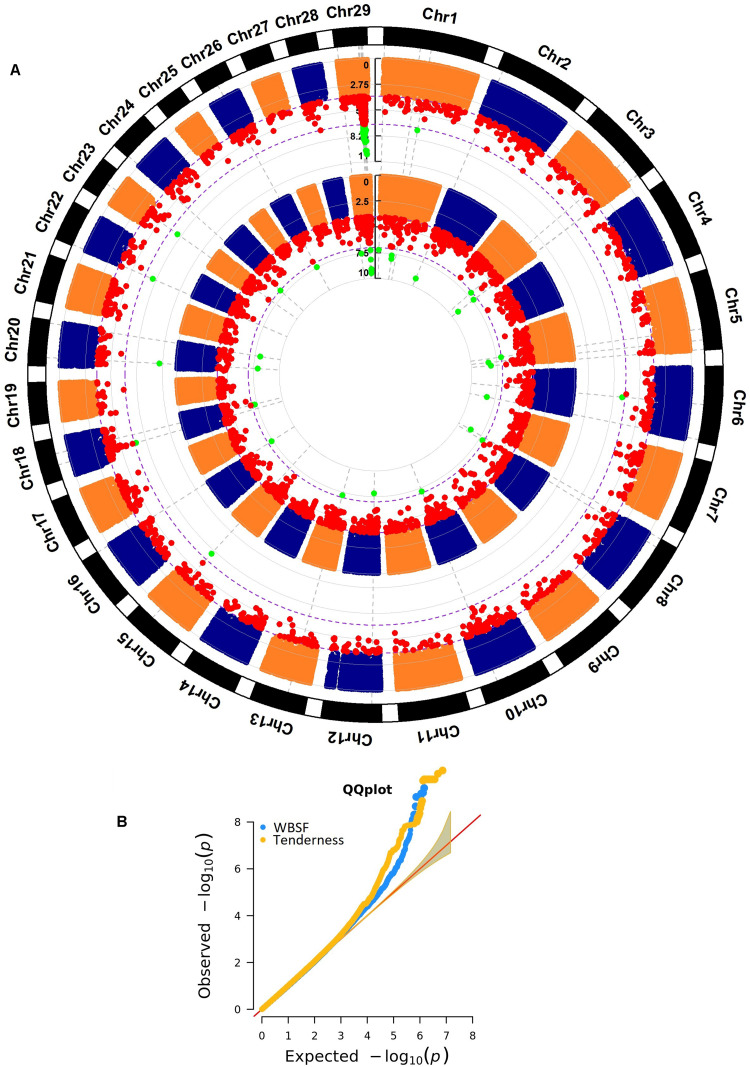
**(A)** Whole genome association results for WBSF (internal Manhattan plot) and tenderness (external Manhattan plot) in *longissimus dorsi* muscle in an Angus population. The significance thresholds are 1 × 10^– 4^ and 1 × 10^– 7^, respectively. **(B)** Q–Q plot for the whole genome association analyses for WBSF and tenderness. Five highly significant markers for WBSF were excluded from this graph (*p*-value ≤ 1.1 × 10^– 17^) but they are presented in Supplementary Table S1.

Warner-Bratzler shear force had a total of 3,181 significant SNPs (Figure 1 and Supplementary Table S1), and 74% of these SNPs were assigned to a gene using all four marker-gene distance bins (1,325 SNPs) or because the marker was located inside the gene (1,016 SNPs). The remaining 26% of the markers were intergenic. Only nine-percent of the associated polymorphisms were assigned to a gene using the 5 kb bin (276 SNPs). For tenderness, 3,926 polymorphisms were identified as associated (Figure 1 and Supplementary Table S2). Seventy-nine percent of these SNPs were determined as assigned to a gene or as genic markers, and the remaining 21% were intergenic markers. A total of 1,474 (38%) associated markers were located inside a gene, and 310 markers were assigned to a gene using the 5 kb bin.

**FIGURE 2:**
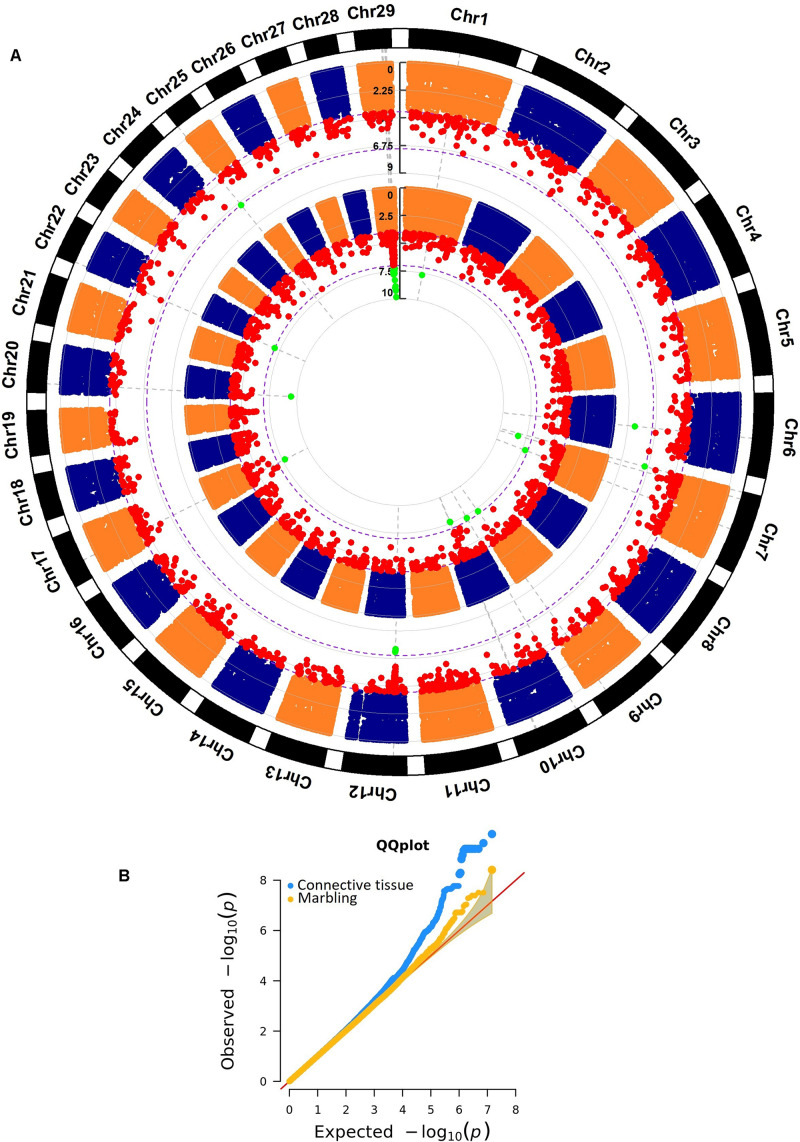
**(A)** Whole genome association results for connective tissue (internal Manhattan plot) and marbling (external Manhattan plot) in *longissimus dorsi* muscle in an Angus population. The significance thresholds are 1 × 10^– 4^ and 1 × 10^– 7^, respectively. **(B)** Q–Q plot for the whole genome association analyses for connective tissue and marbling.

Marbling showed 1,867 significant markers (Figure 2 and Supplementary Table S3). A total of 595 (32%) polymorphisms were located inside a gene (i.e., genic markers), 794 (43%) SNPs were assigned to a gene (i.e., marker located at 5, 10, 50, and 100 kb from a gene), and the remaining associated markers were intergenic.

A total of 3,678 polymorphisms were associated with connective tissue (Figure 2 and Supplementary Table S4). Seventy-nine percent of them were determined as genic polymorphisms or as assigned to a gene, and the remaining 21% were intergenic polymorphisms. Thirty-nine percent of the associated markers were inside a gene (1,450 SNPs), and 7% were assigned to a gene using the 5 kb bin.

**FIGURE 3:**
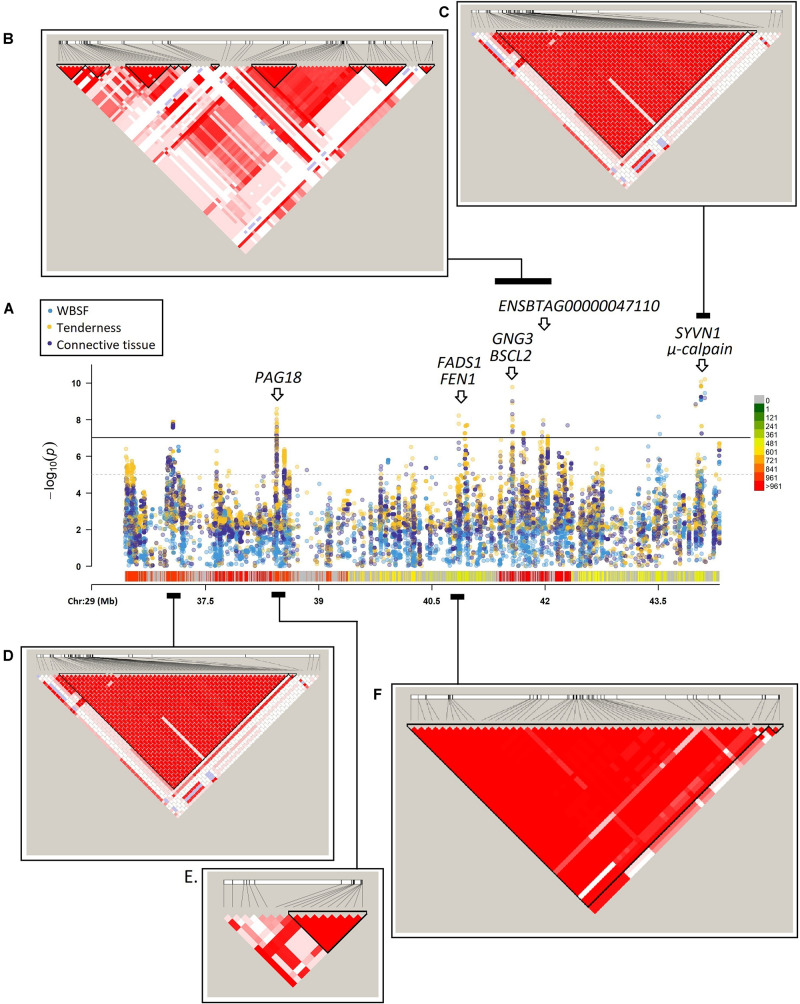
**(A)** Detailed region between the markers on BTA29 located at 36,432,655 and 44,313,046 bp and its association with WBSF, tenderness, and connective tissue. This region harbors 6,100 polymorphisms. The significance threshold used was 1 × 10^– 7^. The white arrows show genes with highly significant genic markers. LD-plots for highly significant subregions comprising: **(B)** *GNG3*, *BSCL2*, and *ENSBTAG00000047110* genes; **(C)** *SYVN1* and μ*-calpain* genes; **(D)** between the base-pair positions 37,062,087 and 37,077,105; **(E)** *PAG18* gene, and **(F)** *FADS1* and *FEN1* genes.

The region located on chromosome 29 between the base-pair positions 36,432,655 and 44,313,046 harbored a total of 6,100 polymorphisms, and 13 of them were highly significant (*p*-value < 1 × 10^–8^) for WBSF, tenderness, and connective tissue. These highly significant markers clustered on five subregions (Figure 3A). The linkage disequilibrium analysis (Figures 3B–F) for these five subregions showed that the region on BTA29 (36,432,655–44,313,046 bp) might harbor multiple QTLs associated with these meat quality traits.

### Enriched Pathway Analysis

Table 2 presents seven pathways identified as enriched in the association analysis for marbling, WBSF, tenderness, and connective tissue. These GO terms were Nucleus (GO:0005634), Cytosol (GO:0005829), and Cytoplasm (GO:0005737). The GO term Nucleus was identified across all four phenotypes ranging from 1.1- to 1.2-fold enrichment and having a total of 201, 188, 187, and 196 associated genes in this pathway for marbling, WBSF, tenderness and connective tissue, respectively.

**TABLE 2:** Pathways identified as enriched for WBSF, tenderness, connective tissue and marbling.

Trait	GO term	Go term name	Sublist 1	Sublist 2	Sublist 3	Sublist 4	*P*-value	Fold
Marbling	GO:0005634	Nucleus	201	1,609	976	6,875	6.3 × 10^–2^	1.1
WBSF	GO:0005634	Nucleus	188	1,622	1,013	6,838	1.6 × 10^–3^	1.2
Tenderness	GO:0005634	Nucleus	187	1,623	976	6,875	6.7 × 10^–3^	1.17
Tenderness	GO:0005829	Cytosol	96	807	1,067	7,691	9.3 × 10^–2^	1.13
Connective tissue	GO:0005634	Nucleus	196	1,613	1,057	6,795	1.3 × 10^–3^	1.2
Connective tissue	GO:0005829	Cytosol	103	800	1,150	7,608	7.6 × 10^–2^	1.14
Connective tissue	GO:0005737	Cytoplasm	210	1,542	1,043	6,866	9.6 × 10^–2^	1.08

*These phenotypes were recorded in an Angus population. Sublist 1 = Genes in genelist in pathway; Sublist 2 = Genes out of genelist in pathway; Sublist 3 = Genes in genelist out of pathway; Sublist 4 = Genes out of genelist out of pathway.*

### Protein–Protein Interaction Network

**FIGURE 4:**
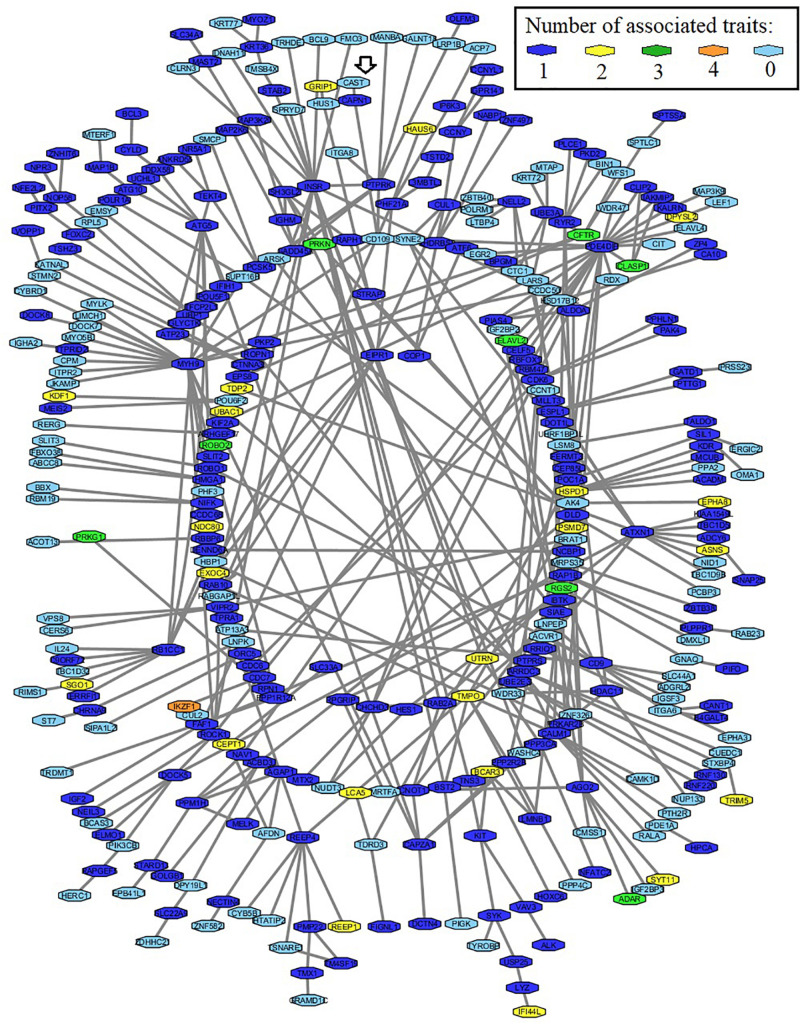
Protein–protein interaction network, including 260 associated genes for marbling, WBSF, tenderness, and connective tissue, and 89 additional non-associated genes (light blue nodes). The phenotypic data was recorded on an Angus population. The arrow shows the μ*-calpain*-*CAST* system.

For this analysis, the query was a list with 589 genes associated with marbling, WBSF, tenderness, or connective tissue. The final network included a total of 260 genes (Figure 4). This network also included 89 non-associated genes since they have physical interaction with some of the associated genes. The total number of interactions described for the network was 431 for all 349 genes. The Calpain-Calpastatin system was included in this network. A number of genes were identified as associated for at least three phenotypes. Some of these genes, such as CF Transmembrane Conductance Regulator (*CFTR*), Cytoplasmic Linker Associated Protein 1 (*CLASP1*), Parkin RBR E3 Ubiquitin Protein Ligase (*PRKN*), Roundabout Guidance Receptor 2 (*ROBO2*) encode structural proteins. The IKAROS Family Zinc Finger 1 (*IKZF1*) which encodes a transcription factor and was identified simultaneously across all association analysis.

### Candidate Structural Protein Assessment of Proteolysis

The 42 most important genes based on significance that encode structural proteins such as integral components of plasma or organelle membranes, cytoskeletal proteins, and organelle matrix-associated proteins were included in the analysis; 81% of these proteins were potential μ-Calpain substrates (Table 3). Some of these structural associated genes and potential substrates of μ-Calpain are *RAB28*, Member RAS Oncogene Family (*RAB28*), Leucine Rich Repeat And Fibronectin Type III Domain Containing 5 (*LRFN5*), Kelch Like Family Member (*5KLHL5*), Integrin Subunit Alpha 2b (*ITGA2B*), Citron Rho-Interacting Serine/Threonine Kinase (*CIT*).

**TABLE 3:** Structural protein genes identified as potential μ-calpain substrates.

Gene stable ID	Associated structure	Accession	Protein detection*
ENSBTAG00000000570	Membrane	XP_027386432.1	
ENSBTAG00000001164	Mitochondrion	XP_027422568.1	
ENSBTAG00000001301	Membrane	XP_027418635.1	
ENSBTAG00000003218	Cytoskeleton	AAI04593.1	Yes
ENSBTAG00000003684	Membrane	XP_027376638.1	Yes
ENSBTAG00000006044	Cytoskeleton	XP_027400083.1	Yes
ENSBTAG00000007116	Membrane	AJK29674.1	
ENSBTAG00000007439	Membrane	XP_027387890.1	
ENSBTAG00000008165	Membrane	AAI47904.1	Yes
ENSBTAG00000008575	Cytoskeleton	XP_027409705.1	
ENSBTAG00000008963	Membrane	AAI23443.1	Yes
ENSBTAG00000009112	Membrane	XP_027379807.1	
ENSBTAG00000009665	Membrane	XP_027407567.1	Yes
ENSBTAG00000010256	Membrane	XP_027380881.1	
ENSBTAG00000011225	Membrane	XP_027409543.1	Yes
ENSBTAG00000012265	Membrane	XP_024847109.1	Yes
ENSBTAG00000012601	Cytoskeleton	NP_001033158.1	Yes
ENSBTAG00000014365	Membrane	XP_027403827.1	
ENSBTAG00000014764	Membrane	NP_776325.1	Yes
ENSBTAG00000014861	Membrane	XP_027386031.1	Yes
ENSBTAG00000015381	Membrane	AAI49810.1	
ENSBTAG00000019252	Cytoskeleton	XP_027375187.1	Yes
ENSBTAG00000019474	Membrane	XP_019824261.1	
ENSBTAG00000019676	Membrane	NP_776535.1	
ENSBTAG00000020444	Cytoskeleton	XP_027393201.1	
ENSBTAG00000021003	Membrane	XP_027381813.1	
ENSBTAG00000021953	Membrane	XP_027397651.1	
ENSBTAG00000025450	Membrane	XP_027410081.1	
ENSBTAG00000026148	Membrane	XP_027403827.1	
ENSBTAG00000031178	Membrane	XP_027399923.1	
ENSBTAG00000037907	Membrane	XP_027381441.1	
ENSBTAG00000039974	Membrane	XP_024840990.1	
ENSBTAG00000044010	Membrane	XP_027375842.1	
ENSBTAG00000046409	Membrane	XP_027386590.1	

Forty-two genes that encode structural proteins were included in the initial analysis. *Protein detection in muscle, adipose tissue or connective tissue (www.genecards.org).

## Discussion

### Recorded Phenotypes

The average intramuscular fat deposition (marbling) score was modest (5.97), but animals with extreme marbling were present (Traces and Abundant marbling deposition). The mean for tenderness and connective tissue was in the upper half between slightly and moderately tender (5.80) and traces and slight amount for connective tissue (5.88). Some individuals had very tender (7.38) and moderately tough (3.00) meat, as well as meat with practically none (7.25) and slightly abundant (3.13) connective tissue content. The mean WBSF was 3.53 kg, 0.77 kg less than the value reported by Leal-Gutiérrez et al. (2019) in a multibreed Brahman-Angus population. The latter population also had less marbling content, and was determined as tougher and with higher connective tissue.

### Association Analysis for Meat Quality Related Phenotypes

In the current study, the associated genes grouped into 3 mechanisms of action: structural proteins, energy metabolism, and cancer-like processes. Leal-Gutiérrez et al. (2019, 2018b) identified similar types of genes in an Angus-Brahman population as associated with meat quality traits. Most of the associated structural proteins are integral components of organelle or plasma membrane, and they could be key anchoring molecules allowing physical interaction between these membranes and the cytoskeleton structure. Several structural protein-coding genes were previously reported as associated with meat quality or differentially expressed when comparing extreme meat quality phenotypes, and some of them are potassium-sodium pumps, cell-cell adhesion-related proteins, collagens and integrins (Lee et al., 2013; Chen L. et al., 2015; Bongiorni et al., 2016; Xia et al., 2016; Zhu et al., 2017). These structural proteins could also be crucial in adipocyte cellular compartmentalization and adipocyte size adaptation. Cancer-like genes could be involved in myocyte and adipocyte proliferation, having a direct effect on meat tenderness and marbling. Seven of the associated regions (Supplementary Table S5) were previously identified in diverse populations as associated with meat quality related traits^2^.

#### Intergenic Regions and Gene Deserts Associated With Meat Quality

A total of 2,908 intergenic regions were identified as associated with meat quality traits (Supplementary Tables S1–S4). The intergenic polymorphisms located at BTA2 (40,929,797 bp), BTA20 (13,250,104 bp), and BTA22 (2,462,415 bp) were highly associated with two or more phenotypes, and the first two markers were on gene deserts. Two gene desert harboring BTA16 (80,477,517 bp) and BTA17 (14,038,252 bp) markers were previously associated with shear force and marbling (Saatchi et al., 2014; Castro et al., 2017), respectively, and included in Supplementary Table S6.

Multiple intergenic polymorphisms were found to explain phenotypic variation in meat quality in cattle. Leal-Gutiérrez et al. (2018b, 2019) performed an association analysis for carcass and meat quality in a multibreed Brahman-Angus population. These analyses identified 195 genomic windows with 68 associated markers. More than 8% of the associated windows and almost 34% of the polymorphisms were intergenic. Out of the 38 intergenic markers and windows detected by Leal-Gutiérrez et al. (2018b, 2019), 21% were located in gene deserts. In the present analysis, 2,908 intergenic markers were identified and the two longest gene deserts were located on BTA26 and BTA5 (931,475 and 1,413,007 bp, respectively) and harbored 40 and 53 associated markers, respectively.

Intergenic associated polymorphisms could regulate genes and pathways by promoting spatial associations with genes (Schierding et al., 2014). This is the case of the human 11q13 locus, which was strongly associated with cancer using GWA analysis. This locus is on a gene desert that harbors multiple regulatory elements and cluster five associated SNPs. Genotypes of the SNP cluster on the 11q13 locus affect transcription factor binding. Upstream Transcription Factor 1 (USF1) and Upstream Transcription Factor 2, C-Fos Interacting (USF2) bind to the common alleles of rs661204 and rs78540526. ETS Transcription Factor ELK4 (ELK4) and GA Binding Protein Transcription Factor Subunit Alpha (GABPA) are specifically bound to the common allele of rs554219. The minor allele of rs75915166 is preferentially bound by GATA Binding Protein 3 (GATA3) (French et al., 2013).

#### Warner-Bratzler Shear Force (WBSF)

Some of the most interesting genes associated with WBSF were μ*-calpain*, *ArfGAP with GTPase Domain, Ankyrin Repeat And PH Domain 1* (*AGAP1*), *Annexin A10* (*ANXA10*), and *Coiled-Coil Domain Containing 80* (*CCDC80*).

A total of three highly significant polymorphisms were identified inside or close to μ*-calpain* for WBSF (BTA29: 44,117,703 bp), tenderness (BTA29: 44,067,968 bp) and connective tissue (BTA29: 44,067,968 bp). The μ-calpain and its inhibitor Calpastatin (CAST) are responsible for postmortem muscle proteolysis, *in vivo* cell viability, and cell proliferation (Geesink, 2005; Geesink et al., 2006; Juszczuk-Kubiak et al., 2008; Van Ba et al., 2015). Leal-Gutiérrez et al. (2019) did not identify an association between μ*-calpain* and meat quality in a Brahman-Angus population; however, Wright et al. (2018) reported an association between Desmin (DES) and Titin (TTN) degradation and μ-calpain autolysis using a subset of individuals from the same population. This discrepancy could be due to the fact that protein analysis might be able to capture variation related to other components involved in μ-calpain activation and activity which is not captured at the DNA level. Multiple polymorphisms in the μ*-calpain*-*CAST* system were associated with meat tenderness in diverse cattle populations (Leal-Gutiérrez and Mateescu, 2019). Although no functional markers have been identified in μ*-calpain*, this gene remains the main candidate gene for meat quality, given its biological role. McClure et al. (2012) reported that at least 1.02 and 1.85% of the observed phenotypic variation in WBSF could be explained by variation in μ*-calpain* and *CAST* in a population composed of five *B. taurus* breeds. Additionally, a μ*-calpain* region harboring 11 markers was highly associated with tenderness and shear force in multiple populations (associated region 7 in Supplementary Table S5). At least three polymorphisms located in this region were highly significant for WBSF, tenderness, and connective tissue in the present population. This region was highly significant in *B. taurus* populations such as Blonde D’aquitaine, Angus, and Charolais (Allais et al., 2014; Mateescu et al., 2017).

One SNP on BTA3 was highly associated with WBSF. This SNP is inside *AGAP1*, a gene expressed in skeletal muscle, bone, and adipose tissue and associated with plasma membrane and Golgi apparatus^3^ (Uhlén et al., 2015). AGAP1 is a GTPase-activating protein for ARF1. ARF1 is involved in lipolysis at the lipid droplets, vesicle trafficking, and cellular morphology. The lipid droplets are a cellular organelle able to store neutral lipids such as triacylglycerol, where they are available to be used as a source of energy and membrane precursor. Dysfunctional ARF1 is responsible for increasing the number of phospholipids available in the lipid droplets, decreasing surface tension, and blocking endoplasmic reticulum (ER) bridge formation (Wilfling et al., 2014). Regulation of ARF1 expression can inhibit extracellular matrix degradation by controlling Matrix Metallopeptidase 9 (MMP9) activity halting invasion in cancerous cells. This process is impaired by inhibiting invadopodia maturation, shedding of membrane-derived microvesicles, and regulation of RHOA and RHOC activity, consequently modifying myosin light-chain (MLC) phosphorylation (Schlienger et al., 2014). Postmortem changes in the phosphorylation of MLC may thus affect the meat texture (Lametsch et al., 2006). The expression of *AGAP1* was also associated with meat quality in a Brahman-Angus population (unpublished data).

On BTA8, a polymorphism mapped to *ANXA10*. The *Annexin* family encodes several cytosolic calcium and membrane-binding proteins. Harder et al. (1997) described that Annexin II (ANXA2), another member of this family, is part of a cholesterol-dependent cytoskeletal complex, including α-Actinin, Ezrin, Moesin, and membrane-associated Actin. Downregulation of *ANXA10* was associated with several types of cancer. Reestablished expression of *ANXA10* in cancerous gastric cells, stalls cell growth, and promotes cellular apoptosis (Kim et al., 2010). Low expression of this gene also promotes liver cell differentiation and halts growth, being linked with malignant phenotype, vascular invasion, and progression (Liu et al., 2002). Leal-Gutiérrez et al. (2019) identified several polymorphisms inside *ANXA10* simultaneously associated with WBSF (two SNPs), marbling (two SNPs), and cooking loss (three SNPs) in a Brahman-Angus population using a methodology designed to identify potential genes with multiple QTLs of medium effect size.

One associated chromosome region on BTA1 harbors *CCDC80*, an extracellular matrix-associated protein. CCDC80, an adipocyte-secreted protein, regulates glucose homeostasis in diet-induced obesity mice. *CCDC80* seems to be linked to obesity-altered secretome in visceral adipose tissue, glucose tolerance imbalances, and associated with chronic inflammation complications (Osorio-Conles et al., 2017). *CCDC80* knockout animals show higher body weight and expansion of white adipose tissue depots. These individuals were even heavier when fed with a high-fat diet, and they also developed impaired glucose metabolism and hepatosteatosis. Primary stromal-vascular cells from *CCDC80*−/− animals exhibited upregulation of genes related to adipogenesis (*CEBPA*, *PPARG*, and *ADIPOQ*) and lipid metabolism (*DGAT1* and *DGAT2*) (Grill et al., 2017).

#### Tenderness

*EMSY Transcriptional Repressor, BRCA2 Interacting* (*EMSY*), *Leucine Rich Repeat Containing 32* (*LRRC32*) and *ENSBTAG00000027438*, *Translocation Associated Membrane Protein 2* (*TRAM2*), *Metallothionein 1E* (*MT1E*), *Integrin Subunit Alpha 2b* (*ITGA2B*) and *Pellino E3 Ubiquitin Protein Ligase Family Member 2* (*PELI2*) genes were associated with tenderness.

One polymorphism on BTA15 was highly associated with tenderness, mapping three genes within the 100 kb bin: *EMSY*, *LRRC32* and *ENSBTAG00000027438*. However, only *EMSY* seems to be biologically related to meat quality. *EMSY* encodes a transcriptional silencing protein highly expressed in skeletal muscle^3^ (Uhlén et al., 2015). This protein is frequently associated with cancer, given that it can promote cell transformation *in vitro*, tumor formation and metastasis *in vivo*. Viré et al. (2014) documented that EMSY represses an antimetastatic microRNA named *miR-31* in cancerous breast samples. Allais et al. (2014) reported this region as associated with shear force in a population composed by Charolais, Limousin, and Blonde d’Aquitaine (associated region 2 in Supplementary Table S5).

*TRAM2*, located on BTA23, was highly associated with tenderness. This gene encodes an integral component of the plasma membrane and a rough endoplasmic reticulum (ER)-associated protein highly expressed in muscle^3^ (Uhlén et al., 2015). TRAM2 is part of a gated channel at the ER membrane. Its upregulation is detectable in oral squamous cell carcinoma cells and promotes metastasis and vascular invasiveness. TRAM2 can control the activity of Matrix Metallopeptidases (MMPs) (Fukushima et al., 2018). The *Bone Morphogenetic Protein* (*BMP*) signaling targets an osteoblast master transcription factor named *RUNX Family Transcription Factor 2* (*RUNX2*), which in turn targets *TRAM2* in osteoblastic cells. The upregulation of *RUNX*2 suppresses *TRAM2* expression, but treatment with BMP2 can activate *TRAM2* expression again. Therefore, *TRAM2* has osteogenic activity (Pregizer et al., 2007). Chen D. et al. (2015) identified *TRAM2* as differentially expressed in a comparison between high-low marbling meat in a crossbreed population composed by Fuzhou Yellow, Limousine, and Wagyu cattle.

One intergenic polymorphism on BTA18 mapped to *MT1E* using the 5 kb bin. This gene was associated with prostate cancer progression and reported as able to enhance tumor proliferation and promote migration in malignant glioma cells by modulating MMPs secretion (Hur et al., 2016). Lee et al. (2013) reported differential expression of *MT1E* when comparing Hanwoo cattle with extreme fat deposition.

*ITGA2B* is on BTA19 and it was associated with tenderness using the 5 kb bin. This gene encodes a plasma membrane-associated protein expressed in bone^3^ (Uhlén et al., 2015). Sasago et al. (2017) identified *ITGA2B* as associated with fatty acid composition in Japanese Black cattle. A polymorphism identified using the 100 kb bin mapped the *PELI2* on BTA10 as associated with tenderness. This gene is expressed in muscle and bone (Uhlén et al., 2015). The E3 ubiquitin ligases family, also described as involved in insulin resistance and diabetes, catalyzes part of the protein degradation performed by the 26S proteasome. Yang et al. (2016) proposed two different mechanisms through which E3 ubiquitin ligases can contribute to insulin resistance. The first mechanism is a direct degradation of key proteins in the insulin signaling pathway such as the insulin receptor. The second mechanism is related to the regulation of pro-inflammatory mediators involved in the same pathway, such as the tumor necrosis factor-α and some interleukins. Yang et al. (2014) identified a pathway supporting the second mechanism. *PELI2* downregulation was present in abdominal adipose tissue from obese people and mice fed with high energy diets as a sign of insulin resistance. This insulin resistance is a consequence of low-level inflammation promoted by inflammatory processes that are modulated by key pro-inflammatory cytokines such as Interleukin 1 Beta (IL1B). Another member of this family, *PELI1*, was identified as differentially expressed in high-marbled meat from Nellore cattle (Simielli Fonseca et al., 2019). Lee et al. (2013) identified *PELI2* as differentially expressed in a comparison between intramuscular fat and omental-subcutaneous fat in Hanwoo cattle.

#### Connective Tissue

*Utrophin* (*UTRN*), *Thioredoxin Related Transmembrane Protein 1* (*TMX1*) and *Transmembrane Protein 170B* (*TMEM170B*) genes were highly associated with connective tissue.

BTA9 (82,770,085 bp) is a genic polymorphism identified in *UTRN*, a *Dystrophin* (*DMD*) *homologous*. This protein is an integral component of the nucleus, cytoskeleton, and plasma membrane, and it is expressed in muscle, adipose tissue, and bone^3^^,^^4^ (Uhlén et al., 2015). UTRN binds to cytoskeletal actin to prevent muscular damage. UTRN and DMD modulate actin filament rotational dynamics promoting resilience (Lin et al., 2012). These homologous proteins are normally present at different subcellular domains; however, when DMD is defective (e.g., Duchenne muscular dystrophy), *UTRN* is upregulated and can compensate for its function. The upregulation of *UTRN* cannot improve neither subsarcolemmal microtubule lattice disorganization nor loss of torque after eccentric contractions (Belanto et al., 2014).

*TMX1* encodes an integral component of the endoplasmic reticulum (ER) membrane mildly expressed in adipose tissue and bone^3^^,^^4^ (Uhlén et al., 2015). This gene is located on BTA10 and was identified using the 100 kb bin. TMX1 localizes at the site of ER-mitochondria Ca^2+^ flux, also named mitochondria-associated membrane (MAM) region. This Ca^2+^ flux from ER to mitochondria regulates mitochondria metabolism; thus, downregulation of *TMX1* associates with reduced ER-mitochondria contacts, faster cytosolic Ca^2+^ clearance, decreased Ca^2+^ flux to mitochondria and repressed mitochondrial metabolism in tumorous tissue (Raturi et al., 2016). ER stress, hypoxia, or short-term nutrient deprivation promotes physical contact between ER and mitochondria (MAM plasticity), being critical for cancer development, neurodegeneration, and metabolic syndrome (Simmen and Herrera-Cruz, 2018). The expression of *TMX1* was associated with meat quality in a multibreed Brahman-Angus population (unpublished data).

A genic marker on BTA23 (44,665,549 bp) mapped to *TMEM170B*. This gene encodes an integral component of the ER and nuclear membrane. *TMEM170B* expression is associated with the overall survival ratio in breast cancer patients. Upregulation of this gene is associated with phosphorylation of cytoplasmic β-catenin, a lower amount of nuclear β-catenin, and changes in the expression of downstream genes (Li et al., 2018). Another member of this family, *TMEM170A*, is involved in ER sheet formation as well as ER shape and morphology. Downregulation of *TMEM170A* produces tubular ER formation, while upregulation was associated with ER sheet formation. Its downregulation also affects nuclear shape and size, density of nuclear pore complexes, and reduction in inner nuclear membrane-associated proteins or their relocalization to the ER (Christodoulou et al., 2016). The expression of *TMEM170B* was associated with meat quality in a multibreed Brahman-Angus population (unpublished data). Other members of this family, such as *TMEM117* and *TMEM236* were previously identified as associated with meat quality traits in cattle (Xia et al., 2016; Zhu et al., 2017).

#### Marbling

*Early Growth Response 2* (*EGR2*), *Ring finger protein 130* (*RNF130*), *C1q, and TNF Related 8* (*C1QTNF8*), SRY-Box 8 (*SOX8*), *Somatostatin Receptor 5* (*SSTR5*), and *Tektin4* (*TEKT4*) and *Solute Carrier Family 20 Member 2* (*SLC20A2*) genes.

The BTA28 (19,057,096 bp) polymorphism mapped to *EGR2* by using the 50 kb bin. This zinc-finger transcription factor promotes cell differentiation, proliferation, and apoptosis. Expression of *EGR2* is low during C3H10T1/2 stem cell progression to adipocyte lineage. On the other hand, the upregulation of this gene allows their progression toward early smooth muscle-like differentiation (Wang et al., 2013). Expression of *EGR1* and *EGR2* correlate with collagen expression during embryonic tendon cell differentiation in limbs. Either of the Early Growth Response proteins can induce *de novo* expression of *Scleraxis BHLH Transcription Factor* (*SCX*), an early identified DNA-binding protein involved in vertebrate tendon formation as well as collagen genes such as *COL1A1*, *COL3A1*, *COL5A1*, *COL12A1*, and *COL14A1*. Mutant *EGR1* and *EGR2* show downregulation of *COL1A1* and a lower number of collagen fibrils in embryonic tendons (Lejard et al., 2011). On BTA7 (1,196,896 bp), a genic SNP mapped *RNF130*. This gene encodes a transcription factor and integral component of membrane expressed in muscle, adipose tissue, and bone^3^ (Uhlén et al., 2015). *RING finger protein 13* (*RNF13*), another member of this family, is an ubiquitin ligase, and it has an N-terminal protease-associated domain and a C-terminal RING finger domain. Ubiquitin ligases are involved in multiple developmental processes, and they are tightly regulated during myogenesis and overexpressed in several tumorous tissues (Jin et al., 2011).

On BTA25 (834,163 bp), an intergenic marker identified using the 50 kb bin, mapped to *C1q, and TNF Related 8* (*C1QTNF8*), SRY-Box 8 (*SOX8*), *Somatostatin Receptor 5* (*SSTR5*), and *Tektin4* (*TEKT4*). *SOX8* encodes a DNA-binding transcription factor involved in developmental processes. The *SOX8* expression promotes the differentiation of articular chondrocytes *in vitro*. Knockdown of *SOX9*, a master transcription factor of chondrogenesis, promotes downregulation of *SOX8*, *COL2A1*, and several chondrogenic related genes (Herlofsen et al., 2014). The upregulation of *SOX8* was identified by Zhang et al. (2014) in Human Hepatocellular Carcinoma; its expression correlates with elevated β-Catenin levels. Chen L. et al. (2015) identified an association between *SOX8* and fatty acid composition in a population composed of purebred, crossbred, and composite Angus individuals. SSTRs encode *G protein-coupled receptors* (*SSTR1-5*), proteins involved in regulation of skin re-epithelialization after injury. SST inhibits cell migration without affecting neither apoptosis nor necrosis. Altered cytoskeleton dynamics associated with delayed lamellipodia formation is present in migrating keratinocytes after treatment with SST. This effect is caused because SST, and its receptors can control the actin cytoskeleton by altering the small GTPase Rac1 availability (Vockel et al., 2011). These proteins are also involved in tumor development. Pisarek et al. (2010) assessed the expression profile of *SST* receptors and identified that in surgically treated human neuroendocrine tumors, *SSTR1* and *SSTR5* were the most highly expressed receptors; therefore, they seem to be key *SST* receptors associated with cell proliferation and migration. *SSTR5* is associated with fatty acid composition in *B. taurus* crossbred animals (Chen L. et al., 2015) and with shear force (associated region 5 in Supplementary Table S5) in a population composed by five *B. taurus* breeds including Angus (McClure et al., 2012). An intergenic polymorphism mapped to *TEKT4* using the 50 kb bin. This gene has mild expression in bone and brain^3^ (Uhlén et al., 2015). Downregulation of *TEKT4* in papillary thyroid cancerous cells *in vitro* stalls proliferation, migration, and colony formation by blocking the PI3K/Akt pathway (Zheng et al., 2018). Some germline variations in *TEKT4* were associated with drug resistance in breast cancer by Jiang et al. (2014). Doublet microtubules are a result of physical interaction between TEKT4 and Tubulin in mammary tissue. Ectopic expression of *TEKT4* causes drug resistance in cancerous tissue, which in turn contributes to microtubule instability, limiting the microtubule-stabilizing effect of this drug. Chen L. et al. (2015) found an association between *TEKT4* and fatty acid composition in *B. taurus* crossbred cattle.

The BTA27 (36,989,414 bp) polymorphism mapped *SLC20A2*, an integral component of plasma membrane expressed in muscle and bone^3^ (Uhlén et al., 2015). Several polymorphisms in *SLC20A2* are responsible for most Primary Familial Brain Calcification cases, a heterogeneous neuropsychiatric disorder. This pathology shows symmetrical and bilateral calcifications more frequently identified in ganglia, thalamus, and cerebellum (Lemos et al., 2015). *SLC20A2* had differential expression for WBSF in a Brahman-Angus population (unpublished data). Aslan et al. (2010) reported a chromosomic region close to this associated polymorphism on ankyrin 1 promoter using a population composed by Angus, Charolais and Limousin as associated with shear force (associated region 6 in Supplementary Table S5).

### Associated Region on BTA29 (36,432,655–44,313,046 bp)

The chromosomal region on BTA29 (36,432,655–44,313,046 bp) harbored multiple markers associated with WBSF, tenderness, and connective tissue (Figure 3A). The LD analysis shows no evidence of historical recombination inside this chromosomic region. All five highly significant subregions seem to belong to different recombinant blocks; therefore, the associated region on BTA29 (36,432,655–44,313,046 bp) might harbor independent QTLs associated with meat quality related traits.

Inside this region there are a number of muscle structure-related genes: *ADAMTS8* (*ADAM Metallopeptidase with Thrombospondin Type 1 Motif 8*), a metallopeptidase activity and integrin-binding coding gene, and the plasma or organelle membrane-associated genes *FADS1* (*Fatty Acid Desaturase 1*), *GNG3* (*G Protein Subunit Gamma 3*), *BSCL2* (*BSCL2 Lipid Droplet Biogenesis Associated, Seipin*), and *SYVN1* (*Synoviolin 1*). *FADS1*, *GNG3*, and *BSCL2* are also involved in fat metabolism. All four plasma or organelle membrane-associated were determined as potential μ*-calpain* substrates by the PROSPER server (Song et al., 2012). Additionally, the proteolytic enzyme responsible for the tenderization process, μ*-calpain*, is located in this region. μ*-calpain* has been extensively analyzed and was found associated with meat quality (Koohmaraie, 1996; Smith et al., 2000; Page et al., 2002; White et al., 2005; Leal-Gutiérrez et al., 2018a, 2019).

*ADAMTS8* is responsible for Linear Morphoea, a connective tissue syndrome in humans associated with thickened skin and subcutaneous tissue, occasionally comprising underlying muscle and bone. Abnormal collagen metabolism, migration, and proliferation are present in affected fibroblasts. These fibroblasts are also less reactive to TGF-β1, reducing myofibroblast differentiation, a process required for skin wound healing, and scarring (Badshah et al., 2018). *BSCL2* is involved in adipogenesis and lipid droplet expansion. Kociucka et al. (2016) reported that during the differentiation of progenitor cells into mature adipocytes, there exists upregulation of *BSCL2* in swine. Knockout *BSCL2* show upregulation of *GPAT* (*Glycerol-3-Phosphate Acyltransferase, Mitochondrial*), and adipogenesis stalling and abnormal lipid droplet morphology. Supersized lipid droplets are present in yeast preadipocytes and fly salivary glands when *GPAT* is upregulated (Pagac et al., 2016). Since adipocyte differentiation couples with changes in cell shape and lipid accumulation, *BSCL2* can have a direct effect on meat quality, especially on marbling.

### Enriched Pathway Analysis

There was enrichment for transcription factors that belong to the GO term GO:0005634 (Nucleus). They drive processes related to muscle, bone, and nervous system development by regulating gene expression. Some of these genes are *EYA Transcriptional Coactivator and Phosphatase 3* (*EYA3*), *Mediator Complex Subunit 12L* (*MED12L*), *GA Binding Protein Transcription Factor Subunit Alpha* (*GABPA*) and *PBX Homeobox 1* (*PBX1*).

*EYAs* are upregulated during embryogenesis and downregulated during development. The upregulation of *EYAs* is present in many tumor types, performing transcriptional cofactor and tyrosine phosphatase functions. Ionescu et al. (2019) found that the upregulation of *EYA3* has proliferative effects. MED12L is part of the Mediator complex, a multiprotein complex involved in transcription of RNA polymerase II-dependent genes. The inclusion of different subunits in the Mediator complex can activate diverse sets of genes transcriptionally. *MED12* was assessed by DeLeon (2015) through a conditional deletion in heart and skeletal muscle in mice. In the heart, *MED12* deletion causes cardiomyopathy, and dysregulation of calcium homeostasis and contractility related genes. In skeletal muscle, severe muscle defects were evident, given that *MED12* is probably involved in muscle growth control. Downregulation of *GABPA* is present in hepatocellular carcinoma tissue and expression of this gene is correlated with tumor grade and distant metastasis. GABPA represses hepatocellular carcinoma cell migration and invasion partly by regulation of CDH1, a cell adhesion protein (Zhang et al., 2017). Gordon et al. (2010) reported that before initiating gene expression, PBX1 and HOXA10 (Homeobox A10) binds to the promoters of osteoblast-related genes, but only HOXA10 remains bound during gene transcription. The ability of PBX1 to bind to histone deacetylases provides its transcription regulation activity.

### Protein–Protein Interaction Network

Out of the 260 associated genes included in the final network, 22 genes were identified simultaneously in two traits, eight other genes as simultaneously associated with three traits and only *IKZF1* (*Ikaros Family Zinc Finger 1*) was found as associated with all four traits simultaneously.

*IKZF1* encodes a zinc finger DNA-binding protein and belongs to the Ikaros gene family. This family is responsible for heritable gene silencing and control of proliferation and lineage determination in the lymphoid system. In hematopoietic cells, Ikaros perform gene activation-inactivation through chromatin remodeling at regions of pericentromeric heterochromatin. Dephosphorylation of Ikaros promotes its binding to the upstream regulatory sequence on *DNTT* (*DNA Nucleotidylexotransferase repressor*), repressing its expression in thymocytes during differentiation (Gurel et al., 2008). CUL2 (Cullin 2), a ubiquitin-protein, and CDC7 (Cell Division Cycle 7), phosphorylases involved in cell cycle and replication, are proteins previously reported as interacting partners of IKZF1. Here, *IKZF1* gene was for the first time associated with meat quality traits, and the role of this gene needs to be ascertained on further investigations.

### Candidate Structural Protein Assessment of Proteolysis

The protein–protein interaction analysis included only two μ-Calpain interacting partners, CAST and CD109. However, through the proteolytic assessment for associated structural proteins, 81% of the analyzed proteins were determined as potential μ-Calpain substrates. Some of these structural proteins are CD9, ITGA2B (Integrin Subunit Alpha 2b), and TMEM170B (Transmembrane Protein 170B), some transmembrane components. These structural proteins are part of the cytoskeletal myocyte framework and might have a direct effect on the tenderization process and meat quality. The amount of lysis of cytoskeletal proteins such as desmin caused by the enzyme μ*-calpain* is an important determinant of the tenderization process; however, transmembrane components can act as membrane anchors for cytoskeletal proteins, being also critical for tenderization (Leal-Gutiérrez et al., 2019).

## Conclusion

A total of 1,867, 3,181, 3,926, and 3,678 associated markers were identified for marbling, WBSF, tenderness, and connective tissue, respectively. Genes involved in mechanisms such as cellular structure, energy metabolism, and cancer-like processes were frequently associated with meat quality. Structural associated proteins are integral components of organelle or plasma membrane. This finding shows that other structural proteins different from filaments, such as Desmin and Nebulin, could have an important role in the tenderization process. Integral components of organelles or plasma membrane might be key anchoring molecules that allow physical interaction between membranes and the cytoskeleton structure. These anchoring proteins can also be important for adipocyte cellular compartmentalization and adipocyte size adaptation since some transmembrane proteins can modulate fat deposition. Some energy metabolism-related genes were also found, and these genes could drive energy storage as fat. The cancer-like genes identified are frequently transcription factors previously associated with cellular proliferation and gene expression modulation. Enrichment for transcription factors that belong to the GO term GO:0005634 (Nucleus) was evident. Around 26% of the associated markers were intergenic polymorphisms, and 13 of these markers were in a gene desert. Intergenic associated polymorphisms and markers in genes deserts could regulate genes and pathways by promoting spatial associations. Some of the most important associated genes were μ*-calpain*, *ANXA10*, *CCDC80*, *LRRC32*, *TRAM2*, *ITGA2B*, *UTRN*, *TMX1*, *TMEM170B*, *SLC20A2*, AGAP1, *ITGA2B*, *PELI2*, *SSTR5*, *EMSY*, *EGR2*, *IKZF1*, and *SOX8*.

An associated region on BTA29 (36,432,655–44,313,046 bp) was highly associated with WBSF, tenderness, and connective tissue and harbored several independent LD-blocks. This region harbors the structural protein-encoding genes *ADAMTS8*, *FADS1*, *GNG3*, *BSCL2 and SYVN1*, and μ*-calpain*, the enzyme responsible for the *post-mortem* tenderization process. *FADS1*, *GNG3*, and *BSCL2* are also involved in fat metabolism.

Overall, this integrative genomic study unravels genetic variants, genes and mechanisms of action affecting meat quality in beef cattle. These findings may contribute to the development of novel genomic strategies for improving meat quality traits via marker-assisted selection.

## Data Availability Statement

The data has been uploaded and is available on the Open Science Framework site at https://osf.io/9cwak/? view_only=4230f697f27d44d2b609820cbbd0e138.

## Ethics Statement

The animal study was reviewed and approved by the Iowa State University and Oklahoma State University Institutional Review Boards.

## Author Contributions

JL-G performed additional analysis and drafted the manuscript. FR performed the association analysis and assisted with manuscript preparation. JR and LK performed imputation to whole genome sequence and assisted with manuscript preparation. FP assisted with the data analyses and manuscript preparation. RM conceived the study and assisted with the data analyses and manuscript preparation. All authors contributed to the article and approved the submitted version.

## Conflict of Interest

The authors declare that the research was conducted in the absence of any commercial or financial relationships that could be construed as a potential conflict of interest.

## Footnotes

1 https://github.com/cbkmephisto/SNPipeline

2 www.animalgenome.org

3 www.proteinatlas.org

4 www.genecards.org

## Supplementary Material

TABLE S1 Association results for WBSF. Gene annotation for the *Bos taurus* UMD 3.1.1 assembly was used. SNPs were assigned to genes using the following criterion: the marker was determined as genic if it was located inside a gene; if the marker was not inside a gene, it was assigned to a gene using the following four marker-gene bin distances: 5, 10, 50, and 100 kb. Otherwise, the marker was determined as a intergenic polymorphism.

TABLE S2 Association results for tenderness. Gene annotation for the *B. taurus* UMD 3.1.1 assembly was used. SNPs were assigned to genes using the following criterion: the marker was determined as genic if it was located inside a gene; if the marker was not inside a gene, it was assigned to a gene using the following four marker-gene bin distances: 5, 10, 50, and 100 kb. Otherwise, the marker was determined as a intergenic polymorphism.

TABLE S3 Association results for marbling. Gene annotation for the *B. taurus* UMD 3.1.1 assembly was used. SNPs were assigned to genes using the following criterion: the marker was determined as genic if it was located inside a gene; if the marker was not inside a gene, it was assigned to a gene using the following four marker-gene bin distances: 5, 10, 50, and 100 kb. Otherwise, the marker was determined as a intergenic polymorphism.

TABLE S4 Association results for connective tissue. Gene annotation for the *B. taurus* UMD 3.1.1 assembly was used. SNPs were assigned to genes using the following criterion: the marker was determined as genic if it was located inside a gene; if the marker was not inside a gene, it was assigned to a gene using the following four marker-gene bin distances: 5, 10, 50, and 100 kb. Otherwise, the marker was determined as a intergenic polymorphism.

TABLE S5 Regions associated with marbling, WBSF, tenderness and connective tissue, and previously reported as associated with meat quality in www.animalgenome.org. Bold polymorphisms were identified as associated with meat quality on the present assay. Position 1 and position 2 present the location of the associated window.

TABLE S6 Intergenic associated polymorphisms classified as gene deserts. Gene annotation for the *B. taurus* UMD 3.1.1 assembly was used. A gene desert was assumed as a >500 kb nucleotide genomic region where no protein coding genes are described. The distance between the associated polymorphism and both adjacent genes must be higher than 200 kb.
